# Comparison of mortality hazard ratios associated with health behaviours in Canada and the United States: a population-based linked health survey study

**DOI:** 10.1186/s12889-022-12849-y

**Published:** 2022-03-10

**Authors:** Stacey Fisher, Carol Bennett, Deirdre Hennessy, Philippe Finès, Mahsa Jessri, Anan Bader Eddeen, John Frank, Tony Robertson, Monica Taljaard, Laura C. Rosella, Claudia Sanmartin, Prabhat Jha, Alastair Leyland, Douglas G. Manuel

**Affiliations:** 1 Ottawa Hospital Research Institute, Ottawa, Ontario Canada; 2 ICES, Ottawa and Toronto, Ontario Canada; 3 School of Epidemiology and Public Health, University of Ottawa, Ottawa, Ontario Canada; 4 Statistics Canada, Ottawa, Ontario Canada; 5 Usher Institute of Population Health Sciences and Informatics, University of Edinburgh, Edinburgh, United Kingdom; 6 Centre for Public Health and Population Health Research, Faculty of Health Sciences & Sport, University of Stirling, Stirling, Scotland; 7 Dalla Lana School of Public Health, University of Toronto, Toronto, Canada; 8 St Michael’s Hospital, University of Toronto, Toronto, Canada; 9 MRC/CSO Social and Public Health Sciences Unit, University of Glasgow, Glasgow, United Kingdom; 10 Department of Family Medicine, University of Ottawa, Ottawa, Ontario Canada

**Keywords:** Population health, Health behaviours, Health surveillance, National health surveys

## Abstract

**Background:**

Modern health surveillance and planning requires an understanding of how preventable risk factors impact population health, and how these effects vary between populations. In this study, we compare how smoking, alcohol consumption, diet and physical activity are associated with all-cause mortality in Canada and the United States using comparable individual-level, linked population health survey data and identical model specifications.

**Methods:**

The Canadian Community Health Survey (CCHS) (2003–2007) and the United States National Health Interview Survey (NHIS) (2000, 2005) linked to individual-level mortality outcomes with follow up to December 31, 2011 were used. Consistent variable definitions were used to estimate country-specific mortality hazard ratios with sex-specific Cox proportional hazard models, including smoking, alcohol, diet and physical activity, sociodemographic indicators and proximal factors including disease history.

**Results:**

A total of 296,407 respondents and 1,813,884 million person-years of follow-up from the CCHS and 58,232 respondents and 497,909 person-years from the NHIS were included. Absolute mortality risk among those with a ‘healthy profile’ was higher in the United States compared to Canada, especially among women. Adjusted mortality hazard ratios associated with health behaviours were generally of similar magnitude and direction but often stronger in Canada.

**Conclusion:**

Even when methodological and population differences are minimal, the association of health behaviours and mortality can vary across populations. It is therefore important to be cautious of between-study variation when aggregating relative effect estimates from differing populations, and when using external effect estimates for population health research and policy development.

**Supplementary Information:**

The online version contains supplementary material available at 10.1186/s12889-022-12849-y.

## Background

Health risk behaviours, including smoking, excessive alcohol consumption, poor diet and lack of physical activity are key contributors to the development of chronic disease and mortality [1]. Modern health surveillance and planning requires an understanding of how these risk factors impact population health. Population health studies requiring estimates of association between modifiable risk factors (such as health behaviours) and disease or mortality may utilize measures of association either directly derived from the population of interest or, if not available, from external epidemiology studies or meta-analyses. However, poor selection of external estimates of association that are not generalizable to the population of interest can lead to invalid results. Generalizability of external estimates can be impaired due to differences in study design, measurement, confounder adjustment, and other sources of methodological heterogeneity. Generalizability can also be impaired by population differences, for example, in age distributions, socioeconomic status, ethnicity, prevalence of comorbidities, and overall baseline health. If the exposure of interest is associated with factors that vary across populations, estimates of association may be different in those populations and thus produce biased estimates when applied to external populations. It is therefore important to understand how study heterogeneity can influence estimated measures of association for population health.

In this study, we compare how smoking, alcohol consumption, diet and physical activity are associated with all-cause mortality in Canada and the United States using individual-level data from comparable, national population health surveys that have been linked to mortality data. To limit methodological heterogeneity, identical model specifications are used to estimate measures of association in each country. We hypothesize that although Canada and the United States are both wealthy, developed countries that are culturally and economically similar, the magnitude of the mortality hazard ratios associated with health behaviours in these two countries will differ.

Many studies have compared the effects of the social determinants of health on population health outcomes in Canada and the United States. For example, income inequality has been strongly associated with mortality in the United States but not in Canada [2]. Health disparities are larger among immigrants to the United States compared to immigrants to Canada [3, 4]. Racial inequalities in health are generally smaller in Canada compared to the United States [4, 5], with larger black-white and Hispanic-white inequalities in the United States, and larger aboriginal-white inequalities in Canada [6]. These studies suggest that the relationships between social determinants of health and population health outcomes are context dependent and that the observed differences in effect may be related to Canada-United States differences in the distribution of and access to social and economic resources. We expect that the effects of health behaviours on mortality are also context dependent, and therefore hypothesize that we will observe differing effects of the health behaviours of interest on mortality in Canada and the United States in the present study.

## Methods

The purpose of this population-based linked health survey study was to estimate and compare the effects of smoking, alcohol consumption, diet and physical activity on all-cause mortality in Canada and the United States.

### Study population and data

Respondent data from the 2003, 2005 and 2007/2008 Canadian Community Health Survey (CCHS cycles 2.1, 3.1 and 4.1) and the 2000 and 2005 United States National Health Interview Survey (NHIS) were used to evaluate and compare adjusted relative estimates of health behaviour hazard ratios in Canada and the United States. Survey cycles were selected considering availability of the health behaviour topics of interest, linkage to mortality data, and survey year. These population health surveys have comparable purposes, designs, sampling and administration methodologies, target populations, exclusions, and content [7]. Both use a multistage stratified cluster design and were conducted through telephone and in-person interviews. All responses are self-reported. The details of the survey methodologies have been previously published [8–10]. Survey respondents were excluded if they were pregnant or younger than 20 years of age at the time of survey administration.

To ascertain all-cause mortality outcomes, CCHS survey respondents have been linked at the individual-level to the Canadian Mortality Database by Statistics Canada [11], and NHIS respondents to the National Death Index by the National Centre for Health Statistics [12], both with follow-up to December 31, 2011. Linkage rates of the CCHS and NHIS to mortality data are approximately 87 and 94%, respectively [11, 13]. Only respondents who agreed to have their survey responses linked to health administrative mortality data, and were linked successfully, were included [11].

### Health behaviour and covariate definitions

Four health behaviour measures were of primary interest: cigarette smoking, alcohol consumption, diet and physical activity. These measures are described in detail in Table 1 and information about derivation and comparability between countries is available [7]. Analyses were adjusted for sociodemographic indicators including education, years since immigration, and ethnicity to further promote comparability between the two populations. Adjustment for factors including active cancer, heart disease, stroke, diabetes and body mass index (BMI) was performed to consider the role of proximal mediating risk factors and should not be interpreted as confounder adjustments. Addititional file 1: Appendix 1 describes how the variables were defined and modelled. Age was centered on the mean age within each sex and country.

**Table 1:** Health behaviour risk factor definitions^a^

Health Behaviour	Definition
**Smoking**	
Heavy smoker	Current smoker, > 20 cigarettes/day
Light smoker	Current smoker, < 20 cigarettes/day
Former smoker	Former smoker
*Never smoker*	*Never smoker*
**Alcohol**	
Heavy drinker	> 21 drinks/week (males) or > 14 drinks/week (females); or binge drinks (5+ drinks on a single occasion) at least once a week
Moderate drinker	3 to 21 drinks/week (male) or 2 to 14 drinks/week (females)
*Light or non-drinker*	*<* *3 drinks/week (males) or* *<* *2 drinks/week (females)*
**Physical Activity**	Average daily metabolic equivalent of task derived from the previous three months of self-reported leisure physical activity (maximum 10)
**Diet Score**^**b**^	
Fruit and vegetable intake	1 point per daily frequency of fruit and vegetable consumption, excluding fruit juice (maximum 8 points)
High potato intake	- 2 points if daily potato consumption > 7 (males) or > 5 (females) a week
High fruit juice intake	- 2 points per daily frequency of fruit juice consumption greater than once a day (maximum −10 points)

^a^ Reference group indicated by italics

^b^ Diet score = 2 baseline points + summation of points from diet attributes (negative overall scores recoded to 0, resulting in a range from 0 to 10)

Single imputation was used to impute missing independent variable values using regression-based predictive mean matching using the aregImpute function from the Hmisc R package [14]. The imputation model consisted of the full list of independent variables, time to event and censoring variables, and auxiliary variables—that is, variables that are not of interest in the analytical models but may nevertheless be useful in generating imputed values (for example, self-perceived health). The imputation procedure used bootstrapping to approximate the process of drawing predicted values from a full Bayesian predictive distribution.

### Statistical analysis

Descriptive age standardized mortality rates were calculated per 10,000 person-years at risk, stratified by sex and country, using the direct method and Canada as the reference population. Health behaviour hazard ratios were assessed using country and sex-specific Cox proportional hazard models, specified to evaluate time to death over 5 years of follow-up. To assess the impact of confounder and mediation adjustment, three increasingly adjusted models were fit: 1) age and health behaviours only, 2) age, health behaviours and sociodemographic indicators, and 3) age, health behaviours, sociodemographic indicators and proximal factors. To produce age-specific measures of association for each of the risk factors, interaction terms between age and the behavioural and disease risk factors were used. Sensitivity analyses dropping the first year of follow-up were performed for the most highly specified models (model 3). One year absolute mortality risk in the unexposed (all healthy reference characteristics) was estimated by multiplying the baseline hazard rate by 1 year, and assuming a constant hazard over time [15].

For each country, rates of observed deaths by sex and 5 year age group in the cohort were calibrated to official counts available from Statistics Canada’s Canadian Socio-Economic Information Management System (CANSIM) [16] and the World Mortality Database [17]. For each country, sex, and five-year age group, observed death rates were calculated and compared to the official rates from CANSIM (for Canada) and the World Mortality Database (for the United States). A correction factor for each individual was calculated as the average of the official rates over the individual’s follow-up time divided by the observed rate. The number of deaths was adjusted by multiplying the outcome (0 = censored alive or 1 = dead) by the correction factor.

Simple adjusted hazard ratios associated with each health behaviour variable are not directly comparable as age was modelled continuously and centered on its mean within each model. Age-specific hazard ratios from the fully specified models (model 3) were therefore calculated using the age-behaviour interaction terms, holding all other covariates fixed. Ages 45 and 70 were selected as the ages for comparison. Hazard ratios were compared by dividing the adjusted hazard ratio from the United States by the adjusted hazard ratio in Canada. Forest plots were used to summarize and present the differences between the countries. Confidence intervals around these ratios were obtained using Taylor’s exact method [18].

Analyses were conducted using SAS v9.3 and Harrell’s HMisc [14] package of functions in R [19]. Forest plots were created using the metafor [20] package in R.

## Results

The CCHS and NHIS cohorts consist of 296,407 and 58,232 respondents, respectively. Within the 1,813,884 million person-years of CCHS mortality follow-up (median 6.4 years), 19,227 deaths (9675 males and 9552 females) were observed. Within the 497,909 person-years of NHIS mortality follow-up (median 6.5 years), 6341 deaths (2973 males and 3368 females) were observed.

Baseline characteristics of the male and female Canadian and United States study cohorts are presented in Table 2. Compared to Canada, the United States cohorts include a higher proportion of respondents under 50 years of age, fewer former smokers and more never smokers, more light or non-drinkers, lower levels of physical activity, and lower fruit and vegetable consumption. Both cohorts are largely white; in Canada, other or multiple ethnicities make up the second largest group (5%), while the United States cohort contains large proportions of Blacks and Latin Americans. Compared to Canada, the United States cohorts have more heart disease, history of stroke, active cancer and a larger proportion of individuals with a BMI above 35.

**Table 2:** Baseline characteristics of male and female study cohorts^a^

	Males		Females	
Characteristic	United States	Canada	United States	Canada
Total N	25,342	134,524	32,890	161,883
Age				
20 to 34	27.8	22.8	27.0	22.9
35 to 49	31.6	27.8	29.7	24.3
50 to 64	23.0	27.1	21.7	26.6
65 to 79	14.2	18.5	16.0	20.0
80+	3.4	3.8	5.6	6.1
Smoking Status				
Heavy smoker	11.5	10.7	6.8	5.7
Light smoker	13.8	16.1	12.8	16.9
Former smoker	26.8	37.1	18.9	28.4
Never smoker	47.0	35.8	60.8	48.8
Missing	0.9	0.3	1.0	0.3
Alcohol Consumption				
Heavy drinker	10.8	12.2	2.7	3.3
Moderate drinker	18.5	19.9	10.4	14.8
Light or non-drinker	68.3	40.7	85.0	52.7
Missing	2.4	27.2	1.9	29.2
Physical Activity^b^				
Median (IQR)	0.6 (0.0, 2.9)	1.5 (0.5, 3.0)	0.3 (0.0, 1.7)	1.3 (0.5, 2.6)
0	41.3	9.7	45.9	11.7
> 0 to < 1.5	18.9	37.9	23.6	41.3
1.5 to < 3	14.1	24.3	13.8	25.1
> 3	24.5	25.5	15.8	20.7
Missing	1.3	2.6	0.9	1.2
Diet (median (IQR))				
Fruits and vegetables	1.6 (1.0, 2.3)	2.6 (1.7, 4.0)	2.0 (1.2, 2.7)	3.6 (2.4, 5.3)
Missing (%)	17.9	20.9	19.3	19.3
Juice	0.5 (0.1, 1.0)	1.0 (0.1, 1.0)	0.5 (0.1, 1.0)	0.7 (0.1, 1.0)
Missing (%)	13.9	18.9	15.0	17.7
Potato	0.2 (0.1, 0.4)	0.4 (0.1, 0.6)	0.2 (0.1, 0.3)	0.3 (0.1, 0.6)
Missing (%)	5.7	19.0	5.2	17.8
Education				
< High school graduation	22.2	22.1	19.9	22.2
High school graduate	24.1	22.6	29.1	24.1
Post-secondary graduate	52.8	54.1	50.4	52.8
Missing	0.9	1.1	0.6	0.9
Years Since Immigration				
< 5 years	2.7	1.3	2.1	1.2
5 to < 10 years	2.8	1.4	2.6	1.3
10 to < 15 years	2.5	1.2	2.2	1.2
> 15 years	9.2	10.4	9.0	10.1
Non-immigrant	82.5	85.5	83.8	86.0
Missing	0.5	0.2	0.4	0.2
Ethnicity				
White	67.0	89.1	64.7	89.6
Black	12.0	0.9	15.0	0.8
South Asian	0.6	1.3	0.4	1.0
Chinese	0.6	1.4	0.4	1.4
Other Asian	1.6	1.6	1.7	1.6
Latin American	16.6	0.4	16.3	0.4
Other/Multiple	1.6	4.8	1.5	4.9
Missing	0.0	0.4	0.0	0.3
Heart Disease				
Yes	11.0	8.4	10.9	6.8
No	88.8	91.4	88.9	93.0
Missing	0.2	0.2	0.2	0.2
Stroke				
Yes	2.5	1.8	2.9	1.6
No	97.4	98.1	97.0	98.4
Missing	0.1	0.1	0.1	0.1
Cancer				
Yes	6.5	5.6	8.2	7.2
No	93.5	62.4	91.7	60.8
Missing	0.1	31.9	0.1	31.9
Diabetes				
Yes	7.7	7.8	7.5	6.8
No	91.2	92.1	91.4	93.1
Missing	1.1	0.1	1.2	0.1
Body mass index (kg/m^2^)				
Median (IQR)	26.6 (24.2, 29.8)	26.3 (23.9–29.1)	25.7 (22.3, 30.0)	24.8 (22.0–28.8)
> 35	6.6	4.5	9.8	5.9
< 35	91.5	93.9	85.2	88.6
Missing	1.9	1.6	5.0	5.5
Survey Cycle				
CCHS 2.1	–	32.7	–	32.7
CCHS 3.1	–	33.7	–	33.5
CCHS 4.1	–	33.6	–	33.8
NHIS 2000	50.7	–	50.7	–
NHIS 2005	49.3	–	49.3	–

Abbreviations: *CCHS* Canadian Community Health Survey, *IQR* interquartile range, *NHIS* National Health Interview Survey

^a^ Numbers are percentages unless otherwise indicated

^b^ Average daily metabolic equivalent of task values

Crude and age-standardized mortality rates per 10,000 person-years are presented in Addititional file 1: Appendix Table 2 and 3 for males and females, respectively. Age standardized mortality rates are higher in the United States compared to Canada, especially among females (USA: 90.3 (95% confidence interval (CI): 87.4, 93.4); Canada: 57.0 (95% CI: 55.9, 58.2)). Heavy smokers had the highest age-standardized mortality rates, which were comparable across countries for males (USA: 161.6, 95% CI: 142.4, 183.4; Canada: 165.4, 95% CI: 154.7, 177.0), but larger among American, compared to Canadian, females (USA: 200.8 (95% CI: 177.8, 226.9); Canada: 136.2 (95% CI: 125.4, 147.8)).

Appendices 4, 5, 6 and 7 present adjusted mortality hazard ratios associated with the three increasingly adjusted development models and sensitivity analyses from the Canadian male, American male, Canadian female and American female cohorts, respectively. Smoking hazard ratios were attenuated with the addition of sociodemographic and disease exposures to the models. Sensitivity analyses removing the first year of study observation did not result in large changes to effect size.

### Comparison of health behaviour hazard ratios in Canada and the United States

**Fig. 1:**
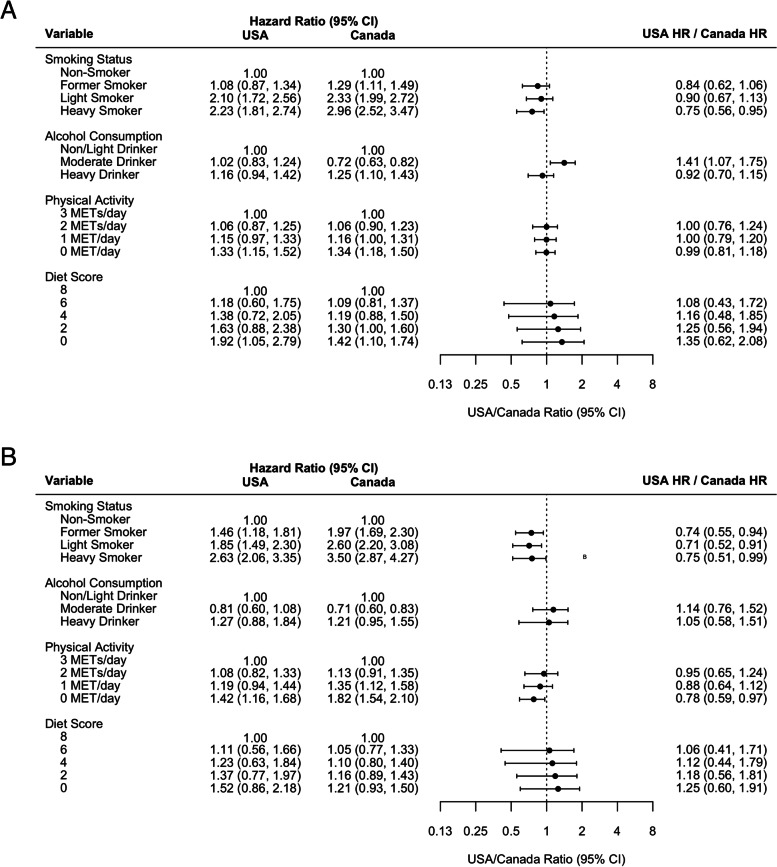
Comparison of the United States and Canadian mortality hazard ratios associated with health behaviours for age 45 (**A**) males and (**B**) females

**Fig. 2:**
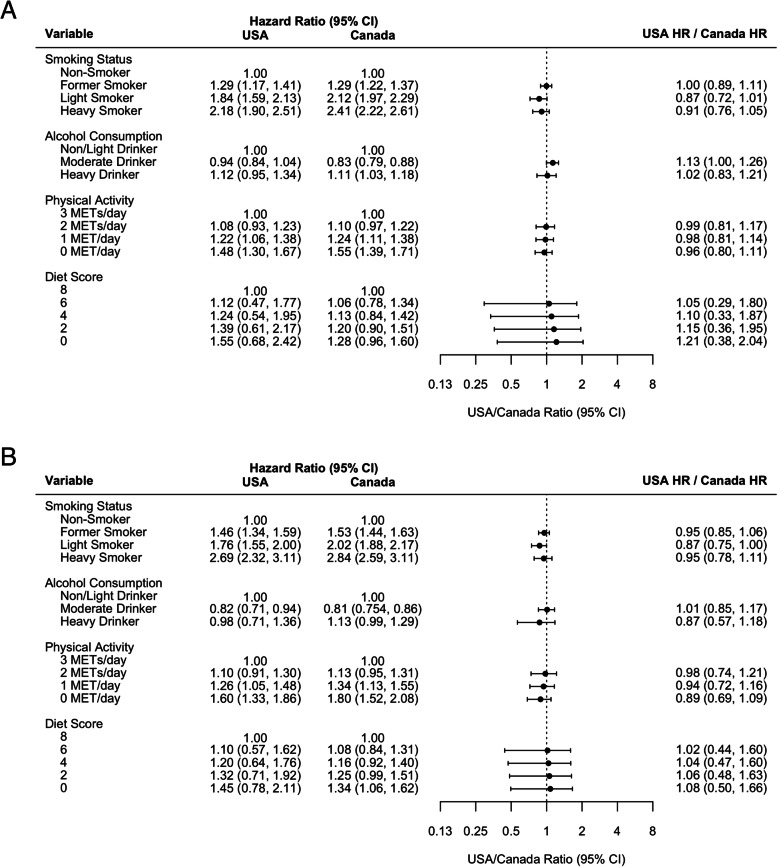
Comparison of the United States and Canadian mortality hazard ratios associated with health behaviours for age 70 (**A**) males and (**B**) females

Due to the presence of continuous age interactions with health behaviours and disease, and to facilitate appropriate comparisons by country (as mean age differs within each country-specific model), we compare adjusted mortality health behaviour hazard ratios from the fully specified United States and Canadian models for age 45 (Fig. 1) and 70 (Fig. 2). Comparison of adjusted hazard ratios associated with all model variables for those age 45 and 70 are reported in Appendices 8 and 9, respectively.

Adjusted mortality hazard ratio estimates associated with heavy smoking, compared to non-smoking, among both males and females 45 years of age are stronger in Canada than in the United States. Among 45 year old males, the hazard ratio in the United States is 0.75 (0.56, 0.95) times the Canadian hazard ratio (Canada: 2.96 (95% CI: 2.52, 3.47); USA: 2.23 (95% CI: 1.81, 2.74); *P* = 0.03). Among 45 year old females, heavy smoking in the United States is 0.75 (95% CI: 0.51, 0.99) times the Canadian estimate (Canada: 3.50 (95% CI: 2.87, 4.27); USA: 2.63 (95% CI: 2.06, 3.35); *P* = 0.05). Hazard ratio point estimates associated with light and former smoking among females 45 years old are also stronger in Canada compared to the United States. Hazard ratio estimates associated with smoking are more comparable between the two countries at age 70.

Adjusted mortality hazard ratio estimates associated with heavy drinking, compared to non/light drinking, are similar in Canada and the United States. Moderate drinking hazard ratio estimates among males differ between the two countries, as moderate drinking was found to be protective in Canada (age 45: 0.72 (95% CI: 0.63, 0.82); age 70: 0.83 (95% CI: 0.79, 0.88)), and not associated with mortality in the United States (age 45: 1.02 (95% CI: 0.83, 1.24); age 70: 0.94 (95% CI: 0.84, 1.04)) (age 45, *P* < 0.01; age 70, *P* = 0.04).

Adjusted hazard ratios associated with physical activity are similar in the two countries, except that 0 metabolic equivalent of task (MET) of daily physical activity, compared to 3 METs, was associated with a stronger hazard ratio among females age 45 in Canada (1.82, 95% CI: 1.54, 2.10) compared to the United States (1.42, 95% CI: 1.16, 1.68) (*P* = 0.04). Diet quality is associated with similar mortality hazard ratios in Canada and the United States among males and females age 45 and 70.

### Comparison of absolute mortality risks

Estimated 1 year mortality risk among those 45 years of age with all healthy reference characteristics is lower for Canadian males (75 (95% CI: 62, 89) per 10,000) than American males (135 (95% CI: 92, 179) per 10,000), and for Canadian females (40 (95% CI: 33, 47) per 10,000) compared to American females (101 (95% CI: 70, 131) per 10,000). Among those 70 years of age, estimated 1 year mortality risk is lower for Canadian males (852, 95% CI: 768, 936 per 10,000) than American males (1003 (95% CI: 803, 1203) per 10,000). One year mortality risk for 70 year old Canadian females is lower (454 (95% CI: 421, 488) per 10,000) than American females (813 (95% CI: 695, 930) per 10,000).

## Discussion

In this study, we compare how smoking, alcohol consumption, diet and physical activity are associated with all-cause mortality in Canada and the United States. Adjusted mortality hazard ratios were generally of similar magnitude and direction but were often stronger in Canada, particularly for smoking at younger ages. Among those 45 years of age, adjusted hazard ratios associated with heavy smoking among males and with current or former smoking among females were significantly larger in Canada compared to the United States. Among those 70 years of age, smoking hazards did not differ statistically but point estimates were also consistently larger in Canada. Similarly, adjusted hazard ratios associated with other health behaviours, except diet, were of similar magnitude or larger in Canada. These results are consistent with existing literature of the effects of the social determinants of health on population health outcomes, and with our study hypothesis that the effects of health behaviours on mortality would differ in these two countries.

Although Canada and the United States are similar in many ways, and are arguably the best counterfactual country for each other, health behaviour hazard ratio estimates may differ because they are being modified by mortality risk factors that differ within each population. This likely includes both individual-level and structural factors including differences in social welfare policies and programs, access to health care services, income distribution, employment security, and the overall extent of socioeconomic inequality [4, 21]. Low income, education and unemployment are associated with a larger health disadvantage in the United States that in Canada [22–24], and within the lowest income quintile and at lower levels of education, Canadians are healthier than their American counterparts [25]. Canada is also known for having a strong emphasis on primary care – avoidable mortality rates are lower in Canada than in the United States, especially for public health and primary care-relevant conditions [26]. These factors may be contributing to an imbalance of effect modifiers of health behaviour hazard ratios on mortality risk between Canada and the United States in this study.

The country-specific underlying mortality risks may also explain why the hazard ratio estimates are more often stronger in Canada. The underlying risk of mortality is the population average absolute risk of death in the absence of observed risk factors and considers all measured and unmeasured study participant characteristics in addition to factors associated with the health environment, such as health care system factors and exposure to air pollution. The present study reports underlying risk as ther 1 year risk of mortality among the unexposed with a ‘healthy profile’ (i.e. all reference characteristics). Underlying mortality risks were estimated to be lower in Canada compared to the United States in both males and females. This can lead to stronger mortality hazard ratios associated with poor health behaviours by leaving more ‘risk space’ in which the harmful effects of the behaviour can act. In other words, these hazard ratios may be compressed by factors contributing to high mortality risk in the United States. Consider a 100 year old individual; assuming their risk of death is greater than 50% in the next year, mathematically, their relative risk of smoking must be less than 2.0 (probably much lower). As their risk of death is already high, the effect of picking up smoking will do little to increase their absolute risk of death. Compared to a younger individual, there is little ‘risk space’ in which the harmful effects of smoking can act. This statistical tendency, or heuristic mathematical rule, has been described by others in the context of health disparity measurement [27, 28]. Relative inequalities are associated with absolute risk such that when the underlying risk is lower, relative inequalities tend to be larger. It is therefore recommended that both relative and absolute risks are reported to provide proper context for inequality interpretation [27, 29], however, few articles do so [30].

Other studies of health behaviours have also found this ‘risk space’ effect. Lear et al. [31] report a stronger protective effect of physical activity on mortality in high and upper-middle income countries compared to lower-middle and low income countries; physical inactivity was associated with higher mortality risk in higher income countries, where underlying risk is generally low, compared to lower income countries, where underlying risk is higher. This effect has also been reported across sex and age [32–34]. In the present study, country-specific adjusted hazard ratios associated with smoking are larger in women compared to men, consistent with the lower underlying mortality risk in women, and are also often larger in the young compared to the old. This effect has generally not be found among groups that vary by socioeconomic status [35, 36], instead finding that the effects of unhealthy behaviours are often stronger in high underlying risk, low socioeconomic status groups. This is likely due to confounding by other factors strongly associated with socioeconomic status such as home ownership, and incomplete adjustment for race, immigration, education and income.

Although the United States and Canada are similar in many ways, we still see measurable differences in the effect of the health behaviours of interest. These results are not likely due to differences in measurement or sampling, as it has been shown that these health behaviours, especially smoking, are ascertained similarly in the CCHS and the NHIS, and that these surveys use very similar sampling methods [7–10]. Effect variation in Canada and the United States has also been reported for social factors using data from the Joint Canada/United States Survey of Health 2002/03 [4, 37, 38] which used identical methodology in both countries [3, 5, 25, 38].

Variation in the effects of health behaviours due to population differences and differences in the underlying risk of mortality raises concerns about how the effects of health behaviours are reported and summarized in the population health literature. Meta-analyses that aggregate health behaviour relative effect estimates from differing populations need to be cautious about assuming that between-study variation is not due to true differences – population heterogeneity can lead to differences in hazard ratios even when methodological heterogeneity is minimized and countries are similar, as demonstrated in the current study. For example, a 2018 meta-analysis by the Global Burden of Disease Study summarized 3992 relative risk estimates from 592 studies to produce dose-response relative risk curves for alcohol consumption and 23 outcomes [39]. The included studies were highly heterogeneous, including populations that vary culturally, socioeconomically and with regards to baseline health, in addition to having substantial methodological differences. The true effects of alcohol within each study population are therefore very likely to vary, even in the absence of methodological differences.

The largest strength of the current study is the use of individual-level and nationally representative data from both countries that allowed for the use of identical model specification and analyses. This reduces the potential for methodological differences, allowing for observed variation to be attributed to population differences with more confidence. However, there is potential for measurement error as there are some differences in the way the CCHS and NHIS collect alcohol, physical activity and diet information [7] and all of the health behaviours explored are time-dependant. Additionally, we did not consider joint effects of health behaviours – for example, heavy alcohol use may strengthen the harmful effects of smoking. Although the reported hazard ratios may therefore be more extreme, we would expect this in both countries. Canadian hazard ratio estimates may also be larger if smokers in Canada smoke more cigarettes on average per day and/or have a longer smoking history, or if heavy drinkers in Canada drink more on average than those in the United States. Although this could explain the observed differences in hazard ratios, it also supports our overall thesis – population differences can lead to true variation in relative health behaviour effect estimates that need to be considered before attempting to generalize.

## Conclusion

Even when methodological and population differences are minimal, the association of health behaviours and mortality can vary across populations. It is therefore important to be cautious of assuming that between-study variation is not due to true differences when aggregating relative effect estimates from differing populations, and when using external effect estimates for population health research and policy development.

## Supplementary Information

**Additional file 1.**

## Data Availability

CCHS data linked to mortality are available for use by approved researchers at Statistics Canada and at Research Data Centre sites [40]; for more information, contact [statcan.had-das-das-had.statcan@canada.ca]. Linked NHIS public use data files are available for download from the CDC website [41].

## Abbreviations

BMI: Body mass index
CANSIM: Canadian Socio-Economic Information Management System
CCHS: Canadian Community Health Survey
CI: Confidence interval
MET: Metabolic equivalent of task
NHIS: National Health Interview Survey
