# Supplementary material for: Comparison of mortality hazard ratios associated with health behaviours in Canada and the United States: a population-based linked health survey study
Source: BMC Public Health. 2022 Mar 10;22:478. doi: 10.1186/s12889-022-12849-y (PMC8915535; doi:10.1186/s12889-022-12849-y)

**Web Materials**

**Appendix 1.** Description of model variables

**Appendix 2.** Male crude and age standardized mortality rates per 10,000 person-years

**Appendix 3.** Female crude and age standardized mortality rates per 10,000 person-years

**Appendix 4.** Male model development; Canada

**Appendix 5.** Male model development; United States

**Appendix 6.** Female model development; Canada

**Appendix 7.** Female model development; United States

**Appendix 8.** Comparison of the United States and Canadian mortality hazard ratios associated with all model variables for age 45 (A) males and (B) females

**Appendix 9.** Comparison of the United States and Canadian mortality hazard ratios associated with all model variables for age 70 (A) males and (B) females

**Appendix 1.** Description of model variables

| Variable Group | Type | Model Definition^1^ | Interaction with Age |
| --- | --- | --- | --- |
| Age^2^ | Continuous | Continuous variable^3^ and linear piecewise spline  =0 if male age <65 or female age<80  = (age - 65) if male >65  = (age -80) if female >80 | - |
| Smoking^4^ | Categorical | Heavy smoker  Light smoker  Former smoker  *Never smoker* | ✓ |
| Alcohol^4^ | Categorical | Heavy drinker  Moderate drinker  *Light or non-drinker* | ✓ |
| Physical Activity^4^ | Continuous | Linear  =log(1+ Average Daily METs^5^) | ✓ |
| Diet Score^4^ | Continuous | Linear | ✓ |
| Education | Categorical | < High school education  High school graduate  *Post-secondary graduate* | 🗴 |
| Immigration | Categorical | > 15 years  10 to < 15 years  5 to < 10 years  < 5 years  *Non-immigrant* | 🗴 |
| Ethnicity | Categorical | *White*, Black, South Asian, Chinese, Other Asian, Latin American, other/multiple | 🗴 |
| Heart Disease | Categorical | Has heart disease  *No heart disease* | 🗴 |
| Stroke | Categorical | Has had a stroke  *No stroke* | 🗴 |
| Cancer | Categorical | Has active cancer  *No active cancer* | ✓ |
| Diabetes | Categorical | Has diabetes  *No diabetes* | ✓ |
| Body Mass Index | Categorical | Linear piecewise spline  =0 if BMI <35  = (BMI – 35) if BMI >35 | 🗴 |

^1^ Reference group indicated by italics

^2^ Age was centered on mean; CCHS males: 49.79, females; 51.25; NHIS males: 46.83, females: 48.32

^3^ For those in the 85+ group in NHIS, median age among 85+ in Canada is used

^4^ Further defined in Table 1

^5^ Metabolic equivalent of task

**Appendix 2.** Male crude and age standardized mortality rates per 10,000 person-years

|  | **United States** | | | | **Canada** | | | |
| --- | --- | --- | --- | --- | --- | --- | --- | --- |
|  | **Person- years** | **Deaths** | **Crude rate** | **Age standardized rate (95% CI)** | **Person- years** | **Deaths** | **Crude rate** | **Age standardized rate (95% CI)** |
| Total | 215,031 | 2,973 | 138.3 | 92.1 (88.4, 95.9) | 819,453 | 9,675 | 118.1 | 87.9 (86.2, 89.6) |
| Age |  |  |  |  |  |  |  |  |
| 20 to 34 | 43,776 | 54 | 12.3 | - | 149,756 | 102 | 6.8 | - |
| 35 to 49 | 72,088 | 196 | 27.2 | - | 239,021 | 418 | 17.5 | - |
| 50 to 64 | 57,186 | 599 | 104.7 | - | 228,359 | 1,572 | 68.8 | - |
| 65 to 80 | 31,505 | 1033 | 327.9 | - | 155,861 | 3,844 | 246.6 | - |
| 80+ | 10,477 | 1091 | 1041.3 | - | 46,457 | 3,739 | 804.8 | - |
| Smoking Status |  |  |  |  |  |  |  |  |
| Heavy smoker | 26,791 | 431 | 160.9 | 161.6 (142.4, 183.4) | 88,117 | 1,162 | 131.9 | 165.4 (154.7, 177.0) |
| Light smoker | 29,995 | 340 | 113.4 | 135.1 (118.3, 154.2) | 133,261 | 1,276 | 95.8 | 141.1 (133.4, 149.3) |
| Former smoker | 54,865 | 1,379 | 251.3 | 92.8 (85.8, 100.5) | 298,934 | 5,311 | 177.7 | 82.9 (80.6, 85.4) |
| Never smoker | 103,381 | 823 | 79.6 | 63.7 (58.9, 68.8) | 299,140 | 1,926 | 64.4 | 59.4 (56.8, 62.0) |
| Alcohol Consumption |  |  |  |  |  |  |  |  |
| Heavy drinker | 24,340 | 221 | 90.8 | 114.6 (96.5 ,136.0) | 132,319 | 1,050 | 79.4 | 106.1 (99.6, 113.0) |
| Moderate drinker | 40,205 | 462 | 114.9 | 78.6 (70.9 ,87.2) | 209,339 | 1,880 | 89.8 | 71.0 (67.9, 74.3) |
| Light or non-drinker | 150,486 | 2,290 | 152.2 | 92.8 (88.6 ,97.2) | 477,795 | 6,745 | 141.2 | 90.8 (88.6, 93.0) |
| Physical Activity^1^ |  |  |  |  |  |  |  |  |
| 0 | 84,933 | 1,780 | 209.6 | 118.7 (112.5, 125.2) | 75,985 | 2,003 | 263.6 | 145.4 (138.8, 152.3) |
| > 0 to < 1.5 | 40,695 | 492 | 120.9 | 80.3 (72.7, 88.7) | 311,730 | 3,542 | 113.6 | 88.9 (86.0, 91.8) |
| 1.5 to < 3 | 31,065 | 277 | 89.2 | 62.8 (54.9, 71.9) | 201,141 | 1,790 | 89.0 | 70.0 (66.8, 73.2) |
| > 3 | 55,362 | 399 | 72.1 | 64.3 (57.6, 71.7) | 211,684 | 1,467 | 69.3 | 61.5 (58.4, 64.6) |
| Diet Score |  |  |  |  |  |  |  |  |
| 0 to < 2.5 | 42,985 | 666 | 154.9 | 113.0 (103.8, 123.1) | 136,164 | 1,726 | 126.8 | 99.8 (95.3, 104.6) |
| 2.5 to < 5 | 129,928 | 1,688 | 129.9 | 90.2 (85.5, 95.2) | 330,343 | 3,466 | 104.9 | 85.4 (82.7, 88.3) |
| 5 to < 7.5 | 18,853 | 287 | 152.2 | 71.2 (62.0, 81.7) | 142,012 | 1,405 | 98.9 | 72.3 (68.6, 76.2) |
| 7.5 to 10 | 1,492 | 13 | 87.2 | 59.4 (32.1, 110.0) | 38,090 | 316 | 83.0 | 68.1 (61.2, 75.9) |
| Education |  |  |  |  |  |  |  |  |
| < High school graduation | 38,864 | 1,026 | 264.0 | 125.4 (116.3, 135.3) | 176,662 | 4,441 | 251.4 | 112.6 (108.5, 116.8) |
| High school graduate | 61,376 | 857 | 139.6 | 97.6 (90.5, 105.1) | 189,741 | 1,603 | 84.5 | 85.1 (81.2, 89.3) |
| Post-secondary graduate | 114,792 | 1,090 | 95.0 | 73.3 (68.6, 78.4) | 453,050 | 3,631 | 80.1 | 74.7 (72.3, 77.1) |
| Years Since Immigration |  |  |  |  |  |  |  |  |
| < 5 years | 7,254 | 15 | 20.7 | 46.6 (21.2, 102.3) | 11,136 | 8 | 7.2 | 41.4 (12.5, 136.7) |
| 5 to < 10 years | 5,963 | 17 | 28.5 | 34.0 (16.9, 68.2) | 11,524 | 18 | 15.6 | 54.0 (32.7, 89.1) |
| 10 to < 15 years | 5,610 | 27 | 48.1 | 91.1 (54.8, 151.3) | 10,310 | 27 | 26.2 | 41.8 (28.6, 61.1) |
| > 15 years | 19,137 | 255 | 133.3 | 76.9 (66.7, 88.6) | 83,494 | 1,406 | 168.4 | 72.5 (68.6, 76.6) |
| Non-immigrant | 177,068 | 2,659 | 150.2 | 94.6 (90.6, 98.8) | 702,989 | 8,216 | 116.9 | 91.3 (89.4, 93.3) |
| Ethnicity |  |  |  |  |  |  |  |  |
| White | 143,729 | 2,165 | 150.6 | 88.6 (84.4, 93.0) | 734,136 | 9,196 | 125.3 | 88.6 (86.8, 90.4) |
| Black | 25,515 | 390 | 152.9 | 112.1 (100.2, 125.4) | 7,345 | 28 | 38.1 | 74.6 (49.0, 113.6) |
| South Asian | 1,403 | 3 | 21.4 | 31.6 (8.1, 122.4) | 10,843 | 31 | 28.6 | 40.7 (28.7, 57.7) |
| Chinese | 1,316 | 11 | 83.6 | 50.4 (25.0, 101.5) | 11,953 | 55 | 46.0 | 51.8 (40.1, 67.1) |
| Other Asian | 3,496 | 26 | 74.4 | 58.1 (37.9, 89.0) | 12,711 | 41 | 32.3 | 50.0 (36.7, 68.0) |
| Latin American | 36,238 | 323 | 89.1 | 92.6 (81.8, 104.8) | 3,619 | 12 | 33.2 | 74.9 (39.7, 141.2) |
| Other/Multiple | 3,334 | 55 | 164.9 | 135.1 (98.4, 185.4) | 38,845 | 312 | 80.3 | 100.0 (89.5, 111.6) |
| Heart Disease |  |  |  |  |  |  |  |  |
| Yes | 20,403 | 895 | 438.7 | 147.5 (130.7, 166.6) | 62,940 | 2,662 | 422.9 | 146.3 (135.1, 158.4) |
| No | 194,629 | 2,078 | 106.8 | 82.4 (78.5, 86.4) | 756,513 | 7,013 | 92.7 | 79.3 (77.5, 81.1) |
| Stroke |  |  |  |  |  |  |  |  |
| Yes | 4,094 | 309 | 754.8 | 209.1 (173.9, 251.4) | 13,014 | 737 | 566.3 | 193.9 (161.0, 233.6) |
| No | 210,938 | 2,664 | 126.3 | 87.5 (83.8, 91.3) | 806,439 | 8,938 | 110.8 | 85.1 (83.3, 86.8) |
| Cancer |  |  |  |  |  |  |  |  |
| Yes | 11,601 | 564 | 486.2 | 130.7 (108.7, 157.2) | 242,171 | 4,685 | 193.5 | 100.3 (97.2, 103.4) |
| No | 203,430 | 2,409 | 118.4 | 89.5 (85.5, 93.6) | 577,283 | 4,990 | 86.4 | 79.9 (77.8, 82.2) |
| Diabetes |  |  |  |  |  |  |  |  |
| Yes | 14,585 | 579 | 397.0 | 151.4 (133.0, 172.3) | 58,735 | 1,937 | 329.8 | 147.0 (136.3, 158.6) |
| No | 200,447 | 2,394 | 119.4 | 85.5 (81.7, 89.5) | 760,718 | 7,738 | 101.7 | 81.5 (79.8, 83.3) |
| Body mass index (kg/m^2^) |  |  |  |  |  |  |  |  |
| > 35 | 14,342 | 184 | 128.3 | 124.5 (102.9, 150.6) | 36,842 | 441 | 119.7 | 116.3 (105.0, 128.9) |
| < 35 | 200,690 | 2,789 | 139.0 | 90.4 (86.7, 94.3) | 782,612 | 9,234 | 118.0 | 86.5 (84.7, 88.2) |

^1^ Average daily METs

**Appendix 3.** Female crude and age standardized mortality rates per 10,000 person-years

|  | **United States** | | | | **Canada** | | | |
| --- | --- | --- | --- | --- | --- | --- | --- | --- |
|  | **Person- years** | **Deaths** | **Crude rate** | **Age standardized rate (95% CI)** | **Person- years** | **Deaths** | **Crude rate** | **Age standardized rate (95% CI)** |
| Total | 282,878 | 3368 | 119.1 | 90.3 (87.4 ,93.4 ) | 994,431 | 9,552 | 96.1 | 57.0 (55.9, 58.2) |
| Age |  |  |  |  |  |  |  |  |
| 20 to 34 | 54,952 | 40 | 7.3 | - | 236,811 | 109 | 4.6 | - |
| 35 to 49 | 89,694 | 199 | 22.2 | - | 250,149 | 376 | 15.0 | - |
| 50 to 64 | 71,180 | 454 | 63.8 | - | 264,196 | 1,527 | 57.8 | - |
| 65 to 80 | 44,283 | 929 | 209.8 | - | 182,966 | 3,680 | 201.1 | - |
| 80+ | 22,770 | 1746 | 766.8 | - | 60,309 | 3,860 | 640.0 | - |
| Smoking Status |  |  |  |  |  |  |  |  |
| Heavy smoker | 19,928 | 277 | 139.0 | 200.8 (177.8, 226.9) | 57,588 | 662 | 115.0 | 136.2 (125.4, 147.8) |
| Light smoker | 37,203 | 337 | 90.6 | 135.5 (122.6, 149.7) | 170,198 | 1,230 | 72.3 | 89.9 (85.2, 94.9) |
| Former smoker | 52,148 | 936 | 179.5 | 105.8 (98.7, 113.3) | 280,815 | 3,189 | 113.6 | 62.2 (60.1, 64.5) |
| Never smoker | 173,598 | 1,818 | 104.7 | 69.4 (66.2, 72.8) | 485,831 | 4,471 | 92.0 | 41.7 (40.4, 43.1) |
| Alcohol Consumption |  |  |  |  |  |  |  |  |
| Heavy drinker | 7,932 | 54 | 68.1 | 101.8 (78.9, 131.3) | 40,651 | 228 | 56.1 | 72.1 (63.5, 81.9) |
| Moderate drinker | 29,940 | 220 | 73.5 | 68.8 (60.9, 77.9) | 203,746 | 1,142 | 56.1 | 46.5 (44.0, 49.2) |
| Light or non-drinker | 245,007 | 3,094 | 126.3 | 92.2 (89.0, 95.5) | 750,034 | 8,182 | 109.1 | 58.8 (57.2, 60.2) |
| Physical Activity^1^ |  |  |  |  |  |  |  |  |
| 0 | 125,717 | 2,317 | 184.3 | 116.8 (111.9, 121.9) | 111,099 | 2,993 | 269.4 | 99.8 (95.2, 104.6) |
| > 0 to < 1.5 | 68,109 | 548 | 80.5 | 66.7 (61.5, 72.3) | 412,226 | 3,835 | 93.0 | 54.8 (53.0, 56.6) |
| 1.5 to < 3 | 39,782 | 253 | 63.6 | 61.0 (54.3, 68.4) | 252,840 | 1,411 | 55.8 | 40.5 (38.5, 42.6) |
| > 3 | 46,546 | 224 | 48.1 | 55.9 (49.6, 63.0) | 208,092 | 859 | 41.3 | 37.7 (35.4, 40.2) |
| Diet Score |  |  |  |  |  |  |  |  |
| 0 to < 2.5 | 52,332 | 682 | 130.3 | 105.9 (98.5, 113.9) | 93,054 | 1,343 | 144.3 | 72.5 (68.3, 77.0) |
| 2.5 to < 5 | 158,947 | 1,794 | 112.9 | 90.5 (86.4, 94.7) | 329,112 | 3,345 | 101.6 | 60.8 (58.8, 63.0) |
| 5 to < 7.5 | 39,329 | 455 | 115.7 | 71.8 (65.5, 78.6) | 255,429 | 1,880 | 73.6 | 47.6 (45.5, 49.9) |
| 7.5 to 10 | 4,338 | 40 | 92.2 | 74.1 (55.3, 99.2) | 120,127 | 596 | 49.6 | 41.2 (38.3, 44.7) |
| Education |  |  |  |  |  |  |  |  |
| < High school graduation | 54,614 | 1,198 | 219.4 | 121.2 (113.8, 129.0) | 218,230 | 4,673 | 214.1 | 70.8 (67.7, 74.1) |
| High school graduate | 82,095 | 1,144 | 139.4 | 92.4 (87.1, 98.0) | 244,805 | 1,922 | 78.5 | 55.0 (52.6, 57.5) |
| Post-secondary graduate | 146,169 | 1,026 | 70.2 | 72.4 (68.3, 76.7) | 531,396 | 2,957 | 55.6 | 49.8 (48.1, 51.6) |
| Years Since Immigration |  |  |  |  |  |  |  |  |
| < 5 years | 6,369 | 8 | 12.6 | 46.8 (20.1, 108.9) | 12,696 | 12 | 9.5 | 30.2 (15.3, 59.6) |
| 5 to < 10 years | 7,656 | 15 | 19.6 | 64.5 (38.6, 107.7) | 12,755 | 21 | 16.5 | 32.4 (20.7, 50.6) |
| 10 to < 15 years | 6,399 | 17 | 26.6 | 60.4 (34.9, 104.7) | 12,846 | 31 | 24.1 | 40.0 (28.1, 56.9) |
| > 15 years | 25,207 | 248 | 98.4 | 67.5 (59.5, 76.6) | 98,369 | 1,274 | 129.5 | 48.3 (45.2, 51.8) |
| Non-immigrant | 237,247 | 3,080 | 129.8 | 94.1 (90.9, 97.5) | 857,765 | 8,214 | 95.8 | 58.9 (57.6, 60.2) |
| Ethnicity |  |  |  |  |  |  |  |  |
| White | 181,630 | 2,510 | 138.2 | 86.5 (83.1, 90.1) | 894,568 | 9,159 | 102.4 | 57.3 (56.1, 58.5) |
| Black | 42,597 | 513 | 120.4 | 120.9 (111.5, 131.0) | 8,067 | 29 | 35.9 | 42.4 (29.5, 60.9) |
| South Asian | 1,038 | 2 | 19.3 | 82.0 (23.3, 288.1) | 9,878 | 22 | 22.3 | 30.2 (19.9, 45.8) |
| Chinese | 1,238 | 5 | 40.4 | 48.7 (20.4, 116.0) | 13,813 | 32 | 23.2 | 23.1 (16.4, 32.5) |
| Other Asian | 4,705 | 35 | 74.4 | 69.9 (50.7, 96.5) | 15,543 | 42 | 27.0 | 32.0 (23.8, 43.1) |
| Latin American | 47,404 | 263 | 55.5 | 74.8 (67.0, 83.6) | 4,353 | 6 | 13.8 | 42.7 (17.6, 103.5) |
| Other/Multiple | 4,264 | 40 | 97.4 | 201.1 (118.2, 342.1) | 48,209 | 262 | 54.3 | 70.6 (62.7, 79.5) |
| Heart Disease |  |  |  |  |  |  |  |  |
| Yes | 27,476 | 971 | 353.4 | 153.1 (139.9, 167.4) | 63,536 | 2,407 | 378.8 | 121.0 (101.6, 144.0) |
| No | 255,402 | 2,397 | 93.9 | 79.7 (76.7, 82.8) | 930,896 | 7,145 | 76.8 | 51.6 (50.4, 52.8) |
| Stroke |  |  |  |  |  |  |  |  |
| Yes | 6,320 | 399 | 631.3 | 226.0 (190.9, 267.5) | 14,213 | 635 | 446.8 | 143.3 (116.3, 176.5) |
| No | 276,557 | 2,969 | 107.4 | 84.9 (82.0, 88.0) | 980,219 | 8,917 | 91.0 | 55.4 (54.2, 56.6) |
| Cancer |  |  |  |  |  |  |  |  |
| Yes | 20,773 | 694 | 334.1 | 166.0 (142.2, 193.8) | 302,162 | 4,894 | 162.0 | 68.9 (66.7, 71.2) |
| No | 262,105 | 2,674 | 102.0 | 84.0 (81.0, 87.2) | 692,270 | 4,658 | 67.3 | 50.0 (48.6, 51.5) |
| Diabetes |  |  |  |  |  |  |  |  |
| Yes | 18,441 | 675 | 366.0 | 187.7 (170.2, 207.0) | 63,475 | 1,720 | 271.0 | 99.8 (92.8, 107.5) |
| No | 264,437 | 2,693 | 101.8 | 79.3 (76.4, 82.4) | 930,956 | 7,832 | 84.1 | 52.5 (51.3, 53.7) |
| Body mass index (kg/m^2^) |  |  |  |  |  |  |  |  |
| > 35 | 29,207 | 346 | 118.5 | 124.6 (112.8, 137.6) | 61,139 | 576 | 94.2 | 78.0 (72.0, 84.4) |
| < 35 | 253,671 | 3,022 | 119.1 | 86.5 (83.5, 89.6) | 933,292 | 8,976 | 96.2 | 55.8 (54.6, 57.0) |

^1^ Average daily METs

**Appendix 4.** Male model development; Canada

|  | **Hazard Ratio (95% CI)** | | | |
| --- | --- | --- | --- | --- |
|  | **Model 1**  Age & Behaviours | **Model 2**  Model 1 + Socioeconomic Factors | **Model 3**  Model 2 + Proximal Mediators | **Sensitivity Analysis**  Model 3 Without Year 1 of Follow-Up |
| Age | 1.10 (1.09, 1.11) | 1.10 (1.09, 1.10) | 1.11 (1.10, 1.13) | 1.11 (1.10, 1.13) |
| Smoking Status |  |  |  |  |
| Never smoker | 1.00 | 1.00 | 1.00 | 1.00 |
| Former smoker | 1.45 (1.27, 1.64) | 1.36 (1.20, 1.54) | 1.29 (1.13, 1.46) | 1.25 (1.08, 1.44) |
| Light smoker | 2.55 (2.23, 2.92) | 2.37 (2.07, 2.71) | 2.29 (2.00, 2.62) | 2.26 (1.95, 2.63) |
| Heavy smoker | 3.29 (2.87, 3.76) | 2.95 (2.57, 3.38) | 2.84 (2.48, 3.26) | 3.01 (2.60, 3.49) |
| Alcohol Consumption |  |  |  |  |
| Light or non-drinker | 1.00 | 1.00 | 1.00 | 1.00 |
| Moderate drinker | 0.69 (0.61, 0.77) | 0.70 (0.62, 0.78) | 0.74 (0.66, 0.83) | 0.76 (0.67, 0.86) |
| Heavy drinker | 1.16 (1.03, 1.29) | 1.14 (1.02, 1.27) | 1.22 (1.10, 1.37) | 1.29 (1.15, 1.46) |
| Physical Activity^1^ |  |  |  |  |
| 0 | 1.00 | 1.00 | 1.00 | 1.00 |
| 1 | 0.83 (0.79, 0.88) | 0.84 (0.79, 0.88) | 0.85 (0.81, 0.90) | 0.86 (0.82, 0.91) |
| 2 | 0.75 (0.69, 0.81) | 0.75 (0.69, 0.82) | 0.78 (0.71, 0.84) | 0.79 (0.72, 0.87) |
| 3 | 0.69 (0.62, 0.77) | 0.70 (0.63, 0.78) | 0.73 (0.65, 0.81) | 0.75 (0.67, 0.84) |
| Diet Score |  |  |  |  |
| 0 | 1.00 | 1.00 | 1.00 | 1.00 |
| 2 | 0.91 (0.86, 0.95) | 0.93 (0.89, 0.98) | 0.92 (0.88, 0.97) | 0.92 (0.87, 0.97) |
| 4 | 0.82 (0.75, 0.91) | 0.87 (0.79, 0.96) | 0.85 (0.77, 0.94) | 0.84 (0.76, 0.94) |
| 6 | 0.75 (0.65, 0.87) | 0.81 (0.70, 0.94) | 0.78 (0.67, 0.90) | 0.77 (0.66, 0.91) |
| 8 | 0.68 (0.56, 0.83) | 0.76 (0.62, 0.92) | 0.72 (0.59, 0.87) | 0.71 (0.57, 0.88) |
| Education |  |  |  |  |
| Post-secondary graduate |  | 1.00 | 1.00 | 1.00 |
| High school graduate |  | 1.11 (1.00, 1.24) | 1.10 (0.98, 1.23) | 1.07 (0.95, 1.21) |
| < High school graduation |  | 1.39 (1.25, 1.55) | 1.32 (1.19, 1.47) | 1.33 (1.18, 1.49) |
| Years Since Immigration |  |  |  |  |
| Non-immigrant |  | 1.00 | 1.00 | 1.00 |
| < 5 years |  | 0.41 (0.19, 0.87) | 0.44 (0.21, 0.94) | 0.38 (0.16, 0.91) |
| 5 to < 10 years |  | 0.43 (0.21, 0.88) | 0.45 (0.22, 0.92) | 0.37 (0.16, 0.85) |
| 10 to < 15 years |  | 0.61 (0.33, 1.11) | 0.63 (0.34, 1.14) | 0.55 (0.28, 1.10) |
| > 15 years |  | 0.85 (0.72, 1.01) | 0.87 (0.73, 1.04) | 0.88 (0.73, 1.06) |
| Ethnicity |  |  |  |  |
| White |  | 1.00 | 1.00 | 1.00 |
| Black |  | 0.64 (0.31, 1.31) | 0.64 (0.30, 1.31) | 0.49 (0.20, 1.21) |
| South Asian |  | 0.39 (0.17, 0.88) | 0.36 (0.16, 0.80) | 0.46 (0.20, 1.05) |
| Chinese |  | 0.68 (0.37, 1.23) | 0.71 (0.39, 1.28) | 0.81 (0.43, 1.53) |
| Other Asian |  | 0.82 (0.49, 1.36) | 0.82 (0.50, 1.36) | 0.95 (0.55, 1.62) |
| Latin American |  | 1.44 (0.73, 2.85) | 1.43 (0.73, 2.83) | 1.58 (0.79, 3.17) |
| Other/Multiple |  | 1.21 (1.01, 1.43) | 1.15 (0.97, 1.37) | 1.15 (0.95, 1.39) |
| Heart Disease |  |  |  |  |
| No |  |  | 1.00 | 1.00 |
| Yes |  |  | 2.03 (1.77, 2.33) | 2.09 (1.79, 2.42) |
| Stroke |  |  |  |  |
| No |  |  | 1.00 | 1.00 |
| Yes |  |  | 2.24 (1.77, 2.84) | 2.15 (1.65, 2.81) |
| Cancer |  |  |  |  |
| No |  |  | 1.00 | 1.00 |
| Yes |  |  | 1.42 (1.29, 1.55) | 1.26 (1.14, 1.39) |
| Diabetes |  |  |  |  |
| No |  |  | 1.00 | 1.00 |
| Yes |  |  | 2.25 (1.96, 2.58) | 2.32 (2.00, 2.70) |
| Body mass index (kg/m^2^) |  |  |  |  |
| < 35 |  |  | 1.00 | 1.00 |
| > 35 |  |  | 1.00 (0.99, 1.01) | 1.00 (0.99, 1.01) |

^1^Average daily METs

**Appendix 5.** Male model development; United States

|  | **Hazard Ratio (95% CI)** | | | |
| --- | --- | --- | --- | --- |
|  | **Model 1**  Age & Behaviours | **Model 2**  Model 1 + Socioeconomic Factors | **Model 3**  Model 2 + Proximal Mediators | **Sensitivity Analysis**  Model 3 Without Year 1 of Follow-Up |
| Age | 1.08 (1.07, 1.09) | 1.09 (1.08, 1.10) | 1.11 (1.10, 1.13) | 1.11 (1.09, 1.13) |
| Smoking Status |  |  |  |  |
| Never smoker | 1.00 | 1.00 | 1.00 | 1.00 |
| Former smoker | 1.22 (1.00, 1.49) | 1.20 (0.98, 1.47) | 1.10 (0.90, 1.34) | 1.08 (0.87, 1.34) |
| Light smoker | 2.33 (1.93, 2.81) | 2.14 (1.78, 2.59) | 2.08 (1.72, 2.50) | 2.17 (1.77, 2.65) |
| Heavy smoker | 2.43 (2.01, 2.93) | 2.37 (1.95, 2.88) | 2.23 (1.84, 2.72) | 2.28 (1.85, 2.81) |
| Alcohol Consumption |  |  |  |  |
| Light or non-drinker | 1.00 | 1.00 | 1.00 | 1.00 |
| Moderate drinker | 0.89 (0.74, 1.07) | 0.92 (0.76, 1.10) | 1.01 (0.84, 1.22) | 0.98 (0.80, 1.20) |
| Heavy drinker | 1.10 (0.90, 1.33) | 1.05 (0.86, 1.27) | 1.15 (0.95, 1.40) | 1.11 (0.90, 1.37) |
| Physical Activity^1^ |  |  |  |  |
| 0 | 1.00 | 1.00 | 1.00 | 1.00 |
| 1 | 0.82 (0.77, 0.88) | 0.85 (0.79, 0.90) | 0.86 (0.81, 0.92) | 0.88 (0.82, 0.94) |
| 2 | 0.73 (0.66, 0.81) | 0.77 (0.69, 0.85) | 0.79 (0.71, 0.88) | 0.82 (0.73, 0.91) |
| 3 | 0.68 (0.59, 0.77) | 0.72 (0.63, 0.82) | 0.75 (0.65, 0.92) | 0.78 (0.68, 0.89) |
| Diet Score |  |  |  |  |
| 0 | 1.00 | 1.00 | 1.00 | 1.00 |
| 2 | 0.82 (0.74, 0.92) | 0.86 (0.77, 0.96) | 0.85 (0.77, 0.95) | 0.85 (0.76, 0.95) |
| 4 | 0.68 (0.55, 0.84) | 0.74 (0.59, 0.91) | 0.73 (0.59, 0.90) | 0.72 (0.57, 0.90) |
| 6 | 0.56 (0.41, 0.77) | 0.63 (0.46, 0.87) | 0.62 (0.45, 0.86) | 0.61 (0.43, 0.86) |
| 8 | 0.46 (0.30, 0.71) | 0.54 (0.35, 0.84) | 0.53 (0.35, 0.81) | 0.52 (0.33, 0.82) |
| Education |  |  |  |  |
| Post-secondary graduate |  | 1.00 | 1.00 | 1.00 |
| High school graduate |  | 1.11 (0.94, 1.30) | 1.12 (0.95, 1.31) | 1.17 (0.99, 1.40) |
| < High school graduation |  | 1.58 (1.32, 1.89) | 1.49 (1.24, 1.79) | 1.55 (1.27, 1.88) |
| Years Since Immigration |  |  |  |  |
| Non-immigrant |  | 1.00 | 1.00 | 1.00 |
| < 5 years |  | 0.47 (0.28, 0.82) | 0.54 (0.32, 0.94) | 0.44 (0.23, 0.84) |
| 5 to < 10 years |  | 0.57 (0.34, 0.96) | 0.64 (0.38, 1.07) | 0.65 (0.38, 1.13) |
| 10 to < 15 years |  | 0.66 (0.40, 1.08) | 0.79 (0.48, 1.28) | 0.78 (0.46, 1.31) |
| > 15 years |  | 0.80 (0.61, 1.06) | 0.85 (0.65, 1.13) | 0.86 (0.64, 1.15) |
| Ethnicity |  |  |  |  |
| White |  | 1.00 | 1.00 | 1.00 |
| Black |  | 1.45 (1.19, 1.76) | 1.38 (1.14, 1.68) | 1.43 (1.17, 1.76) |
| South Asian |  | 0.39 (0.06, 2.80) | 0.38 (0.05, 2.74) | 0.46 (0.06, 3.32) |
| Chinese |  | 1.57 (0.63, 3.91) | 1.63 (0.66, 4.01) | 1.92 (0.77, 4.80) |
| Other Asian |  | 1.03 (0.52, 2.04) | 1.01 (0.51, 2.00) | 0.87 (0.39, 1.92) |
| Latin American |  | 1.28 (1.01, 1.61) | 1.26 (1.00, 1.59) | 1.29 (1.00, 1.65) |
| Other/Multiple |  | 2.33 (1.64, 3.31) | 2.08 (1.45, 2.97) | 2.11 (1.44, 3.10) |
| Heart Disease |  |  |  |  |
| No |  |  | 1.00 | 1.00 |
| Yes |  |  | 1.95 (1.60, 2.37) | 2.09 (1.70, 2.57) |
| Stroke |  |  |  |  |
| No |  |  | 1.00 | 1.00 |
| Yes |  |  | 2.59 (1.87, 3.58) | 2.62 (1.85, 3.71) |
| Cancer |  |  |  |  |
| No |  |  | 1.00 | 1.00 |
| Yes |  |  | 2.20 (1.68, 2.88) | 1.96 (1.45, 2.65) |
| Diabetes |  |  |  |  |
| No |  |  | 1.00 | 1.00 |
| Yes |  |  | 2.39 (1.94, 2.93) | 2.40 (1.92, 2.99) |
| Body mass index (kg/m^2^) |  |  |  |  |
| < 35 |  |  | 1.00 | 1.00 |
| > 35 |  |  | 1.00 (0.99, 1.01) | 1.00 (0.99, 1.02) |

^1^Average daily METs

**Appendix 6.** Female model development; Canada

|  | **Hazard Ratio (95% CI)** | | | |
| --- | --- | --- | --- | --- |
|  | **Model 1**  Age & Behaviours | **Model 2**  Model 1 + Socioeconomic Factors | **Model 3**  Model 2 + Proximal Mediators | **Sensitivity Analysis**  Model 3 Without Year 1 of Follow-Up |
| Age | 1.10 (1.09, 1.10) | 1.10 (1.09, 1.11) | 1.11 (1.10, 1.13) | 1.12 (1.11, 1.14) |
| Smoking Status |  |  |  |  |
| Never smoker | 1.00 | 1.00 | 1.00 | 1.00 |
| Former smoker | 1.99 (1.75, 2.25) | 1.92 (1.69, 2.18) | 1.85 (1.63, 2.10) | 1.84 (1.60, 2.12) |
| Light smoker | 2.66 (2.33, 3.05) | 2.53 (2.20, 2.90) | 2.44 (2.13, 2.81) | 2.55 (2.20, 2.97) |
| Heavy smoker | 3.79 (3.24, 4.44) | 3.56 (3.03, 4.18) | 3.32 (2.83, 3.90) | 3.42 (2.87, 4.07) |
| Alcohol Consumption |  |  |  |  |
| Light or non-drinker | 1.00 | 1.00 | 1.00 | 1.00 |
| Moderate drinker | 0.68 (0.60, 0.78) | 0.70 (0.61, 0.80) | 0.73 (0.64, 0.84) | 0.76 (0.66, 0.88) |
| Heavy drinker | 1.11 (0.91, 1.36) | 1.11 (0.91, 1.37) | 1.19 (0.97, 1.46) | 1.24 (1.00, 1.54) |
| Physical Activity^1^ |  |  |  |  |
| 0 | 1.00 | 1.00 | 1.00 | 1.00 |
| 1 | 0.71 (0.67, 0.76) | 0.72 (0.68, 0.77) | 0.74 (0.70, 0.79) | 0.78 (0.73, 0.83) |
| 2 | 0.58 (0.53, 0.64) | 0.60 (0.54, 0.66) | 0.62 (0.56, 0.69) | 0.67 (0.60, 0.75) |
| 3 | 0.51 (0.45, 0.57) | 0.52 (0.46, 0.59) | 0.55 (0.48, 0.62) | 0.60 (0.53, 0.69) |
| Diet Score |  |  |  |  |
| 0 | 1.00 | 1.00 | 1.00 | 1.00 |
| 2 | 0.93 (0.89, 0.98) | 0.95 (0.91, 1.00) | 0.95 (0.90, 0.99) | 0.97 (0.92, 1.02) |
| 4 | 0.87 (0.79, 0.96) | 0.91 (0.82, 1.00) | 0.90 (0.81, 0.99) | 0.93 (0.84, 1.03) |
| 6 | 0.81 (0.71, 0.94) | 0.86 (0.75, 0.99) | 0.85 (0.73, 0.98) | 0.90 (0.77, 1.05) |
| 8 | 0.76 (0.63, 0.92) | 0.82 (0.68, 0.99) | 0.80 (0.66, 0.97) | 0.87 (0.71, 1.07) |
| Education |  |  |  |  |
| Post-secondary graduate |  | 1.00 | 1.00 | 1.00 |
| High school graduate |  | 1.04 (0.93, 1.17) | 1.03 (0.91, 1.16) | 1.08 (0.95, 1.23) |
| < High school graduation |  | 1.35 (1.20, 1.52) | 1.25 (1.11, 1.41) | 1.29 (1.13, 1.47) |
| Years Since Immigration |  |  |  |  |
| Non-immigrant |  | 1.00 | 1.00 | 1.00 |
| < 5 years |  | 0.94 (0.52, 1.70) | 0.94 (0.52, 1.71) | 0.88 (0.46, 1.67) |
| 5 to < 10 years |  | 1.03 (0.59, 1.81) | 1.08 (0.61, 1.89) | 0.93 (0.48, 1.79) |
| 10 to < 15 years |  | 0.86 (0.48, 1.54) | 0.88 (0.49, 1.58) | 0.79 (0.41, 1.53) |
| > 15 years |  | 1.01 (0.84, 1.21) | 1.02 (0.85, 1.23) | 0.99 (0.82, 1.21) |
| Ethnicity |  |  |  |  |
| White |  | 1.00 | 1.00 | 1.00 |
| Black |  | 1.31 (0.77, 2.22) | 1.32 (0.77, 2.25) | 1.17 (0.61, 2.21) |
| South Asian |  | 0.94 (0.51, 1.72) | 0.91 (0.50, 1.68) | 1.15 (0.62, 2.13) |
| Chinese |  | 0.60 (0.31, 1.16) | 0.59 (0.30, 1.15) | 0.64 (0.31, 1.33) |
| Other Asian |  | 0.66 (0.37, 1.18) | 0.66 (0.37, 1.19) | 0.71 (0.38, 1.35) |
| Latin American |  | 0.54 (0.17, 1.75) | 0.55 (0.17, 1.79) | 0.72 (0.24, 2.18) |
| Other/Multiple |  | 1.19 (0.99, 1.44) | 1.13 (0.93, 1.36) | 1.07 (0.87, 1.32) |
| Heart Disease |  |  |  |  |
| No |  |  | 1.00 | 1.00 |
| Yes |  |  | 2.04 (1.74, 2.39) | 2.00 (1.68, 2.39) |
| Stroke |  |  |  |  |
| No |  |  | 1.00 | 1.00 |
| Yes |  |  | 2.24 (1.73, 2.92) | 2.15 (1.60, 2.88) |
| Cancer |  |  |  |  |
| No |  |  | 1.00 | 1.00 |
| Yes |  |  | 1.67 (1.52, 1.84) | 1.45 (1.30, 1.61) |
| Diabetes |  |  |  |  |
| No |  |  | 1.00 | 1.00 |
| Yes |  |  | 1.99 (1.71, 2.32) | 2.08 (1.76, 2.45) |
| Body mass index (kg/m^2^) |  |  |  |  |
| < 35 |  |  | 1.00 | 1.00 |
| > 35 |  |  | 0.99 (0.98, 1.00) | 1.00 (0.99, 1.01) |

^1^Average daily METs

**Appendix 7.** Female model development; United States

|  | **Hazard Ratio (95% CI)** | | | |
| --- | --- | --- | --- | --- |
|  | **Model 1**  Age & Behaviours | **Model 2**  Model 1 + Socioeconomic Factors | **Model 3**  Model 2 + Proximal Mediators | **Sensitivity Analysis**  Model 3 Without Year 1 of Follow-Up |
| Age | 1.08 (1.07, 1.09) | 1.09 (1.08, 1.10) | 1.11 (1.10, 1.13) | 1.12 (1.10, 1.14) |
| Smoking Status |  |  |  |  |
| Never smoker | 1.00 | 1.00 | 1.00 | 1.00 |
| Former smoker | 1.64 (1.36, 1.98) | 1.68 (1.39, 2.03) | 1.46 (1.20, 1.78) | 1.41 (1.15, 1.74) |
| Light smoker | 2.07 (1.70, 2.50) | 1.90 (1.56, 2.31) | 1.84 (1.51, 2.24) | 1.84 (1.50, 2.27) |
| Heavy smoker | 2.84 (2.31, 3.49) | 2.82 (2.27, 3.51) | 2.64 (2.12, 3.28) | 2.85 (2.27, 3.57) |
| Alcohol Consumption |  |  |  |  |
| Light or non-drinker | 1.00 | 1.00 | 1.00 | 1.00 |
| Moderate drinker | 0.69 (0.53, 0.89) | 0.73 (0.56, 0.95) | 0.81 (0.62, 1.05) | 0.86 (0.65, 1.12) |
| Heavy drinker | 1.13 (0.81, 1.58) | 1.15 (0.82, 1.61) | 1.23 (0.88, 1.72) | 1.22 (0.86, 1.74) |
| Physical Activity^1^ |  |  |  |  |
| 0 | 1.00 | 1.00 | 1.00 | 1.00 |
| 1 | 0.76 (0.70, 0.82) | 0.79 (0.73, 0.86) | 0.83 (0.77, 0.91) | 0.85 (0.77, 0.92) |
| 2 | 0.64 (0.56, 0.73) | 0.69 (0.60, 0.79) | 0.75 (0.66, 0.85) | 0.77 (0.67, 0.88) |
| 3 | 0.57 (0.49, 0.68) | 0.63 (0.53, 0.74) | 0.69 (0.59, 0.82) | 0.71 (0.60, 0.85) |
| Diet Score |  |  |  |  |
| 0 | 1.00 | 1.00 | 1.00 | 1.00 |
| 2 | 0.88 (0.80, 0.97) | 0.93 (0.84, 1.03) | 0.90 (0.82, 1.00) | 0.92 (0.83, 1.02) |
| 4 | 0.78 (0.64, 0.95) | 0.86 (0.71, 1.05) | 0.81 (0.67, 0.99) | 0.84 (0.68, 1.04) |
| 6 | 0.69 (0.51, 0.92) | 0.80 (0.59, 1.08) | 0.73 (0.55, 0.99) | 0.77 (0.57, 1.06) |
| 8 | 0.60 (0.41, 0.90) | 0.74 (0.50, 1.11) | 0.66 (0.45, 0.98) | 0.71 (0.47, 1.08) |
| Education |  |  |  |  |
| Post-secondary graduate |  | 1.00 | 1.00 | 1.00 |
| High school graduate |  | 1.20 (1.02, 1.42) | 1.19 (1.01, 1.41) | 1.22 (1.02, 1.45) |
| < High school graduation |  | 1.60 (1.33, 1.94) | 1.44 (1.19, 1.75) | 1.46 (1.19, 1.78) |
| Years Since Immigration |  |  |  |  |
| Non-immigrant |  | 1.00 | 1.00 | 1.00 |
| < 5 years |  | 0.53 (0.25, 1.10) | 0.60 (0.29, 1.26) | 0.65 (0.31, 1.38) |
| 5 to < 10 years |  | 0.49 (0.25, 0.97) | 0.58 (0.30, 1.15) | 0.57 (0.28, 1.15) |
| 10 to < 15 years |  | 0.72 (0.41, 1.26) | 0.83 (0.47, 1.45) | 0.82 (0.46, 1.45) |
| > 15 years |  | 0.84 (0.62, 1.13) | 0.93 (0.69, 1.25) | 0.88 (0.63, 1.21) |
| Ethnicity |  |  |  |  |
| White |  | 1.00 | 1.00 | 1.00 |
| Black |  | 1.68 (1.41, 2.00) | 1.59 (1.32, 1.90) | 1.61 (1.33, 1.95) |
| South Asian |  | 0.12 (0.00, 7.49) | 0.14 (0.00, 7.81) | 0.14 (0.00, 8.61) |
| Chinese |  | 1.79 (0.58, 5.49) | 1.92 (0.62, 5.92) | 2.18 (0.70, 6.76) |
| Other Asian |  | 1.68 (0.94, 3.00) | 1.78 (1.00, 3.18) | 1.79 (0.96, 2.32) |
| Latin American |  | 1.12 (0.87, 1.44) | 1.12 (0.87, 1.45) | 1.10 (0.84, 1.44) |
| Other/Multiple |  | 1.29 (0.79, 2.12) | 1.08 (0.65, 1.79) | 1.00 (0.58, 1.74) |
| Heart Disease |  |  |  |  |
| No |  |  | 1.00 | 1.00 |
| Yes |  |  | 1.87 (1.55, 2.27) | 1.79 (1.46, 2.20) |
| Stroke |  |  |  |  |
| No |  |  | 1.00 | 1.00 |
| Yes |  |  | 1.93 (1.41, 2.64) | 1.91 (1.36, 2.68) |
| Cancer |  |  |  |  |
| No |  |  | 1.00 | 1.00 |
| Yes |  |  | 2.45 (2.01, 3.00) | 2.09 (1.67, 2.62) |
| Diabetes |  |  |  |  |
| No |  |  | 1.00 | 1.00 |
| Yes |  |  | 2.76 (2.27, 3.35) | 2.78 (2.26, 3.43) |
| Body mass index (kg/m^2^) |  |  |  |  |
| < 35 |  |  | 1.00 | 1.00 |
| > 35 |  |  | 1.01 (1.00, 1.02) | 1.01 (1.00, 1.02) |

^1^Average daily METs

**Appendix 8.** Comparison of the United States and Canadian mortality hazard ratios associated with all model variables for age 45 (A) males and (B) females

(A)


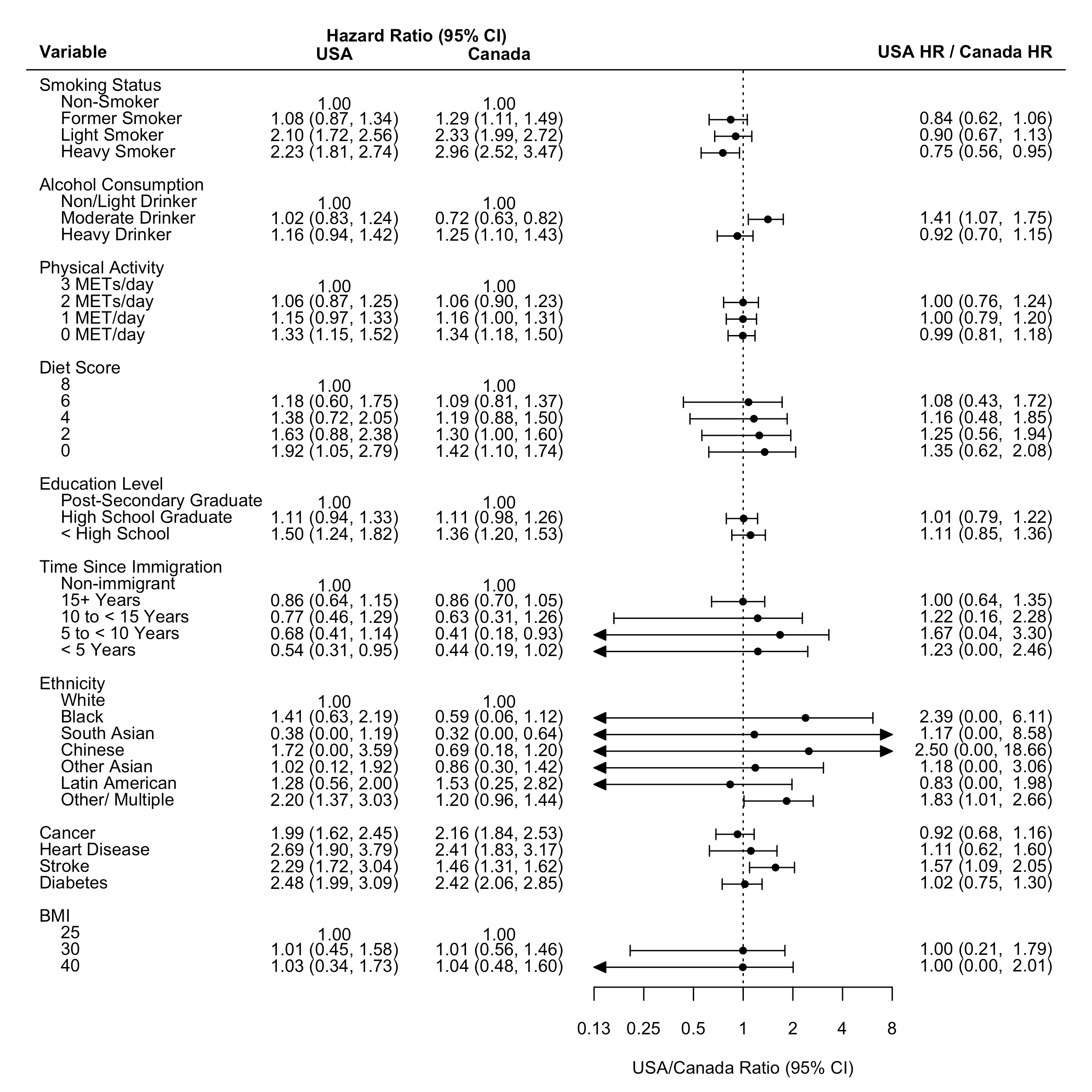


(B)


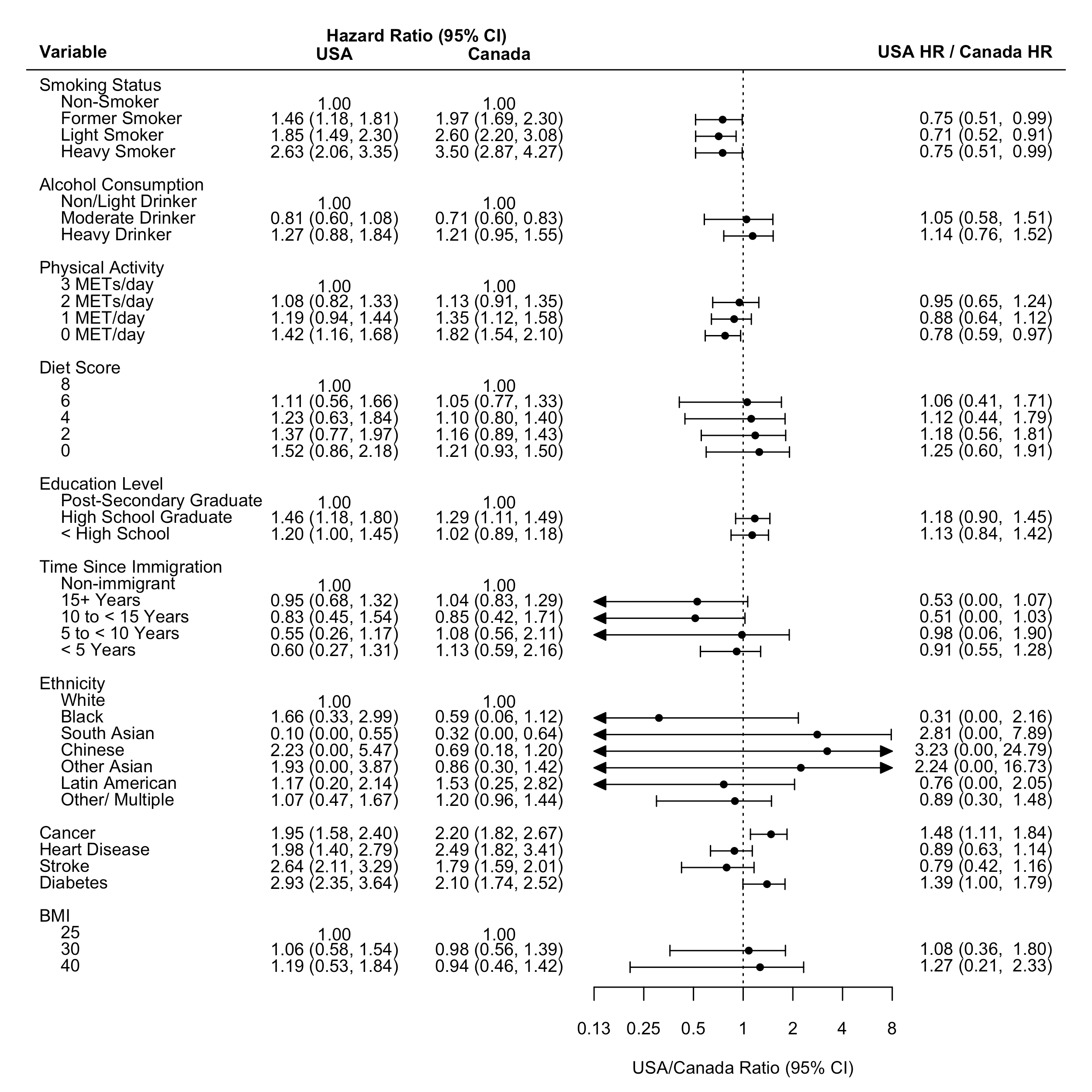


**Appendix 9.** Comparison of the United States and Canadian mortality hazard ratios associated with all model variables for age 70 (A) males and (B) females

(A)


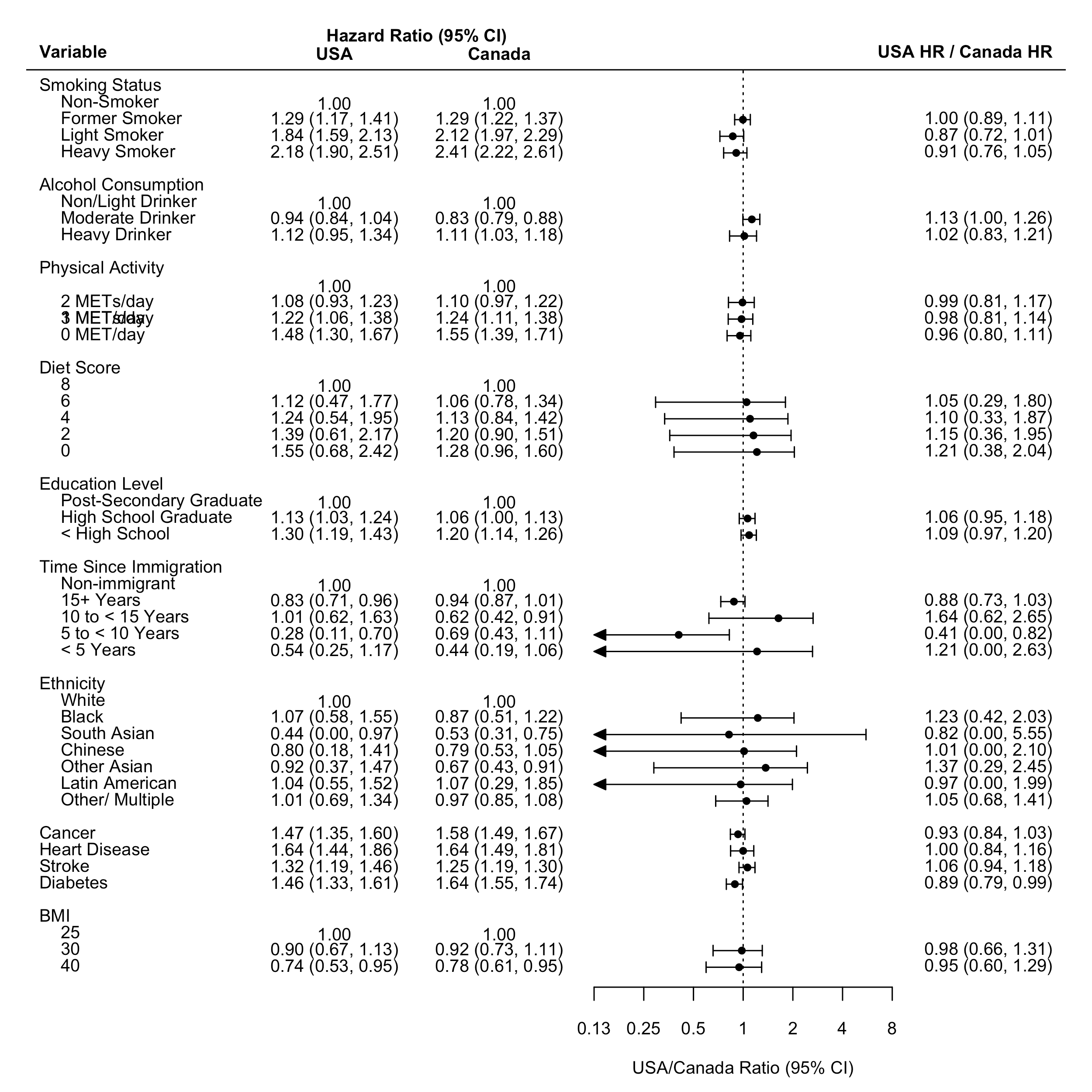


(B)


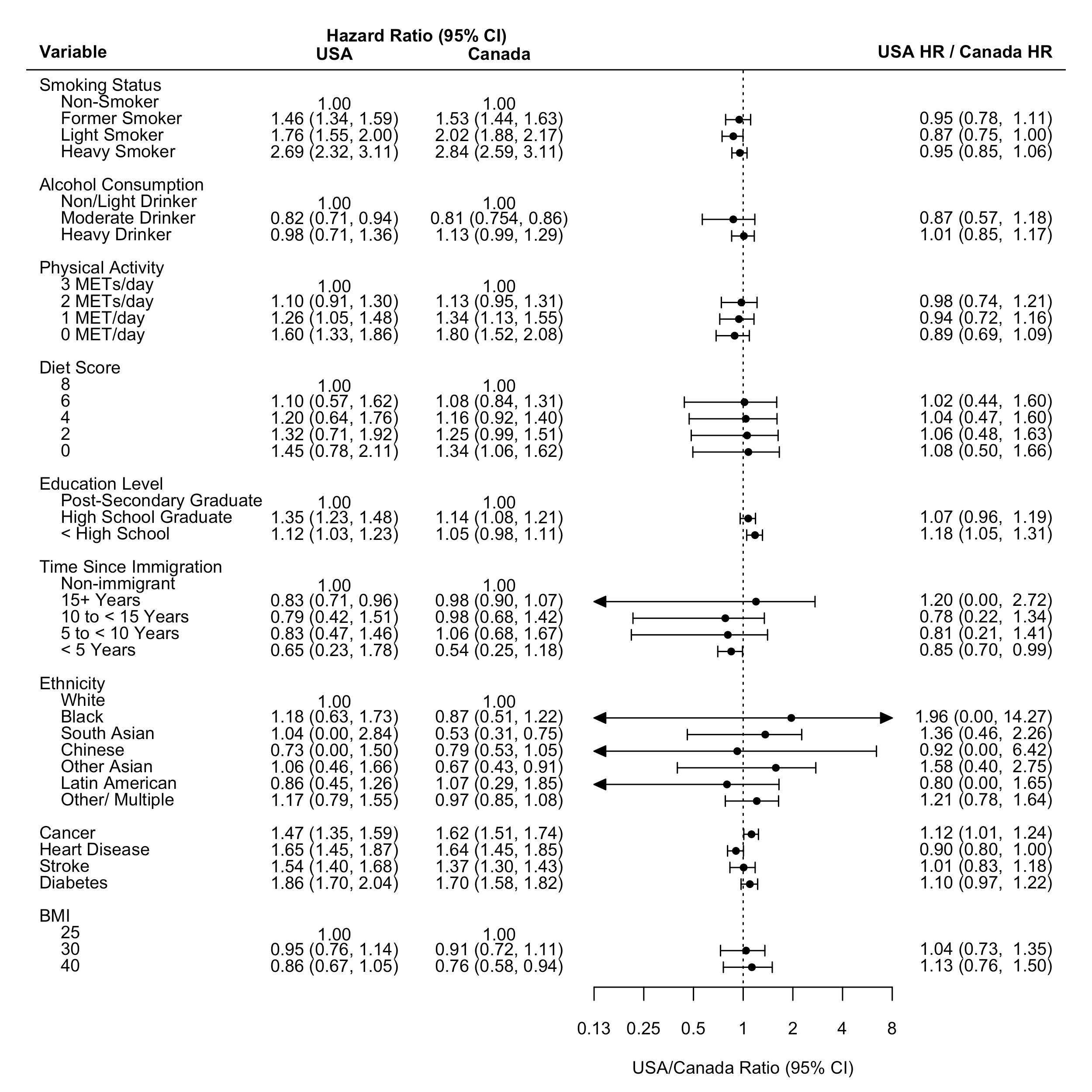

Supplement: Supplementary file 1 — Additional file 1. [file 12889_2022_12849_MOESM1_ESM.docx]
